# A vision of the use of technology in medical education after the COVID-19 pandemic

**DOI:** 10.15694/mep.2020.000049.1

**Published:** 2020-03-26

**Authors:** Poh-Sun Goh, John Sandars

**Affiliations:** 1 National University of Singapore; 2 Edge Hill University Medical School

**Keywords:** technology, medical education, transformative change, coronavirus, COVID-19

## Abstract

This article was migrated. The article was marked as recommended.

Medical education across the world has experienced a major disruptive change as a consequence of the COVID-19 pandemic and technology has been rapidly and innovatively used to maintain teaching and learning. The future of medical education is uncertain after the pandemic resolves but several potential future scenarios are discussed to inform current decision-making about the future provision of teaching and learning. The use of emergent technology for education, such as artificial intelligence for adaptive learning and virtual reality, are highly likely to be essential components of the transformative change and the future of medical education. The benefits and challenges of the use of technology in medical education are discussed with the intention of informing all providers on how the changes after the pandemic can have a positive impact on both educators and students across the world.

## Introduction

The purpose of this Personal View is to offer a vision of the use of technology in medical education after the COVID-19 pandemic begins to resolve. Both authors have a keen interest in the innovative use of technology in medical education and an awareness of the current and future trends in the use of technology to enhance teaching and learning. We will begin by a reflection on the current increased use of technology as a major factor in enabling the continuation of medical education during the pandemic. This reflection will be followed by a discussion of several potential future scenarios that are based on the emergent trends in the use of technology but also an understanding of how complex social systems respond over time to the trigger of major events. We will also discuss the benefits and challenges of the future use of technology in medical education after the pandemic resolves.

A transformative change in the current approach to medical education across the world is inevitable and although the full extent is unknown at the current time it is essential to consider potential future scenarios to begin the process of preparing for the future ( Chermack, 2004). We fully appreciate the difficulty that many medical educators will experience in considering the future at a time when most educators across the world are deeply engaged in responding to the current enormous challenges, both personal and professional as clinicians and educators. However, it is essential that all educational policy makers, curriculum planners and educators across the continuum of medical education, from basic to continuing, can begin to critically reflect on the present situation and make appropriate decisions about the future of medical education for when the pandemic resolves.

## The impact of the COVID-19 pandemic

The pandemic has resulted in the widespread disruption of medical education and professional training ( Ahmed *et al.*, 2020; Murphy, 2020). Examples include reduced teaching with redeployment of medical educators to clinical care and the quarantine and impact of illness on medical educators and students. Measures to ensure social distancing have included closure of medical schools and working from home for both educators and students. Local and international travel, and attendance at training programs has been halted. Physical attendance at workshops and symposia, conferences, clinical attachments and visiting fellowships has ceased. Tragically, there have also been an increasing number of deaths that include doctors and other healthcare professionals.

## The current response to the COVID-19 pandemic

Overall, the current response to the pandemic has been the increased awareness and adoption of currently available technologies in medical education, and also in the wider education sector ( Iwai, 2020). These changes across the continuum of medical education have been mainly to replace existing approaches for the provision of medical education, driven by the urgency to implement a feasible and practical solution to the crises, with educators using familiar technology.

Medical schools and other medical education providers, including commercial organizations and professional bodies, have rapidly scaled up the provision of educational content and training online, as well as faculty development in the use of technology, especially by online courses. Large group in-person lectures have been replaced by streamed online lectures, using technologies for screen capture and online dissemination. Small group sessions and tutorials have been replaced with interactive Webinars using web conferencing platforms. All of these learning resources can be easily accessed from mobile devices.

A major challenge for medical educators at the present time has been to replicate the experience of clinical encounters. These encounters range from clinic and ward rounds to interactive patient sessions to training in interpersonal and inter professional communication and clinical skills. Currently available technology, such as videos, podcasts, simple virtual reality, computer simulations and serious games, are beginning to be used to assist educators and facilitate student learning and training in these areas. Simple online platforms, such as websites and blogs, can provide basic information but also offer opportunities to host videos for demonstrating essential skills, such as procedural clinical skills and communication ( Dong and Goh, 2015). Medical educators can remotely coach students with real time mobile video tools and apps.

The increasing trends of competency based medical education (CBME) and programmatic assessment require regular assessments of student achievement. Medical schools have creatively responded to the challenge of a lack of opportunities to observe student performance or to hold large scale examinations. Formative and summative assessments for core knowledge have started to use a variety of online tools and platforms. The range is from websites, discussions forums and online discussion spaces to real-time online chat and communication apps. Feedback on performance and the assessment of skills acquisition has similarly started to maximize the ubiquitous availability of video and audio on mobile devices to enable assessment in authentic contexts, either clinical or simulated. These assessments should be ideally based on high quality evidence and theory informed assessment and evaluation strategies ( Martin *et al*., 2019).

We are heartened to see greater national collaboration between medical schools to share educational and training resources (PIVOT MedEd, 2020). Commercial providers are also increasing their engagement and collaboration with medical schools.

## The future after the COVID-19 pandemic

We consider that it will be highly unlikely that there will be a return to the previous approach to the provision of medical education as existed before the pandemic, especially the contribution of technology for enhancing teaching and learning. The change will be transformative, with a major change in how individuals and the wider social system within which each individual lives and works. The uncertainty at the current time is around the extent of this transformation since it is dependent on the complex interaction between several major factors that are difficult, and some observers would say almost impossible, to predict. These conversion factors are mainly related to the length of time that the pandemic is disruptive, since a long disruption is likely to produce significant alteration in several of the factors. The factors include the number and availability of educators, economic constraints and the need to rapidly expand the clinical workforce. All of these factors will have a major impact on the future way that educators and their institutions will provide medical education.

### Understanding the transformation

Our framework to understand transformative change is Normalisation Process Theory (NPT). This sociological theoretical framework has been increasingly used to understand how a new practice, such as the use of technology, becomes embedded within a social system (“normalisation”) through an active process, both individually and collectively, that occurs over a period of time ( Scantlebury *et al*., 2017). The new practice becomes embedded when it is routinely incorporated in the everyday work of individuals and groups. The key phases of this dynamic interactive process between individuals and others in the social system begin with the development of a shared understanding of the benefits and importance of the change to be achieved, and this is followed by the building and sustaining of individual and collective commitment around an intervention. Finally, there is ongoing resolution of any issues around differences in opinions about the new practice and there is increased allocation of resources to enable the new practice to become embedded. Once the practice is embedded it is considered both individually and collectively as the usual way of working and the new practice is unlikely to revert back to the original practice, especially if there have been major conversion factors that have initiated the transformation.

The NPT framework suggests at the present time that the process of transformation in the increased use of technology in medical education is within the early phases, with what appears to be a rapid and progressive individual and collective acceptance and commitment to the use of technology to enhance teaching and learning. The extent to which the transformation leads to embedding of technology will be variable across different providers of medical education but one future potential future scenario is that only minor transformative change will occur, with increased use of current technology, especially with a greater emphasis on online learning and mobile devices to replace face to face group teaching and meetings.

However, another potential future scenario is that of major transformative change in medical education, especially if there has been a major disruptive influence on the way that we all live and work after the pandemic resolves. If there is a major disruptive challenge to medical education, such as a vastly reduced number of educators and the need to rapidly expand the education of the future workforce across the continuum of medical education, the variety of current technology being used to augment medical education will be inefficient and inappropriate to meet the high demand. Educators will need to develop and implement innovative solutions in response to this high demand and an awareness of future trends in the use of technology is invaluable in beginning to prepare for the future.

### Understanding the emergent technology

The Horizon 2020 Teaching and Learning report was produced by an expert panel to highlight how emergent technology has the potential to transform future provision of higher education ( Brown *et al*., 2020). There are two main envisaged changes; adaptive learning and extended reality.

The introduction of adaptive learning offers a personalized approach to enable all students to access a wide range of learning resources and to provide information to educators about how students are learning from their experience. Essential for adaptive learning is the integrated application of two types of emergent technology: artificial intelligence (AI) and learning analytics ( Chan and Zary, 2019; Wartman and Combs, 2019). The application of artificial intelligence creates “thinking machines” to provide learning content and assessments that can adaptively interact with students using text and voice. These applications range from learning anatomy to complex clinical diagnostic and management challenges. Robotic tutors that are adaptive to problem-solving have been used alongside school children to facilitate their individual self-regulated learning ( Jones and Castellano, 2018). Learning analytics collect information about the process and outcomes of learning that are essential to inform educators about the progress and trajectory of both individual and groups of students. The learning potential of these new approaches is that students can obtain personalized learning that is tailored to their individual needs and there is also the opportunity to reduce the time for the development of individual competence and to decrease the time required for face to face interaction with educators and patients.

Extended reality (XR) provides students with learning experiences that either blends physical and virtual elements (augmented reality or AR) or provides a totally virtual immersive experience (virtual reality or VR) ( Zweifach and Triola, 2019). The immersive experience has the intention to replicate a real-life experience and this can be delivered through headsets or mobile devices. An emergent trend in technology is haptic simulation which replicates the physical sensations of a real-life experience, such as touch. The learning potential is that these sophisticated experiences can be applied to a range of clinical topics, from communication and clinical skills to deliberate practice of surgical procedures, and also they can be integrated with adaptive learning to realize additional benefits.

### The middle ground future scenario

The potential future scenario for medical education and the contribution of technology to enhance teaching and learning after the resolution of the pandemic is likely to be in the middle ground between the two extreme ends of the spectrum that we have presented in the two previous scenarios. It is highly likely that the use of technology will increase and this also includes an accelerated application of many of the newer types of emergent technology that have been described in the Horizon 2020 report. However, the extent to which these types of emergent technology have become, and continue to be, embedded will be dependent on the complex mix of factors within a particular context. These factors include the length of time of disruption to previous approaches to medical education and the available resources, including support from learning technologists and access to the emergent technology. Overall, an integrated approach that combines elements of both technology and face to face teaching and learning experiences is likely to characterise the future scenario.

## The benefits of change after the COVID-19 pandemic

Whatever the change and extent of transformation in medical education after the pandemic it is inevitable that there will increased individual and collective awareness and acceptance of the innovative potential that technology, including emergent technology, can offer to enhance teaching and learning across the continuum of medical education ( Goh, 2016). The ‘anytime anywhere’ aspect of using technology offers new opportunities for specific groups of students, such as increasing access and participation to part-time students and providing shortened programmes for gifted or talented students.

It will be interesting to see if the current increased spirit of national collaboration of medical educators to freely create, share and curate learning content will continue. There is the exciting opportunity for these collaborations to spread and include educators from across the world. The benefits in meeting the World Health Organisation goals to provide universal health coverage through an urgent and rapid increase in trained workforce cannot be underestimated (World Health Organisation, 2015). However, the digital divide between countries, especially between high and low and middle income countries, is potentially a major challenge to these ventures. Technology that is appropriate to the local contexts, with lower bandwidth cellular and online networks, will need to be considered and international collaboration between medical schools will need to be developed.

## The challenges of change after the COVID-19 pandemic

We have presented several potential future scenarios of the use of technology, including emergent technology, in medical education after the pandemic resolves and our overall vision has been positive, with a discussion of the advantages for teaching and learning. However, it is important to consider the challenges that will need to be addressed if the expected potential transformative changes are to continue to be embedded and further evolve over time.

The effective of use of technology for enhancing teaching and learning has been discussed earlier but achieving the desired outcome and impact will only be realised by continuing to develop all medical educators in how to skillfully align the various contributory factors, including the learner, the learning objectives, the learning content, the instructional design, the technology and the context ( Zaharias and Poylymenakou, 2009). The Horizon 2020 report also highlights the essential need to implement ‘learning engineering’ if an emergent technology, such as more sophisticated virtual reality, is being considered for use in teaching and learning. The components of this approach includes design thinking, agile and iterative development, user experience evaluation and the application of learning science to craft the learning experience ( Badwan *et al.*, 2018). Many educators are likely to require further development and training in the effective use of technology for enhancing teaching and learning.

The development of emergent technology, especially when specifically for teaching and learning, is often costly and requires a range of different expertise. However, the Horizon 2020 report also highlights the increasing trend for open educational resources (OER) that are available without restriction, including financial cost, to both educators and students across the world. We consider that the opportunity for all medical education providers to offer OER has never been more appropriate and we urge all providers to continue their current collaborative ventures.

Finally, at this time of transformative change in the use of technology in medical education, we recommend that the opportunity is grasped to increase the development of an educational scholarship related to the use of technology and to increase the implementation of global benchmarking standards ( Goh and Sandars, 2019). Both of these ventures have the future potential to ensure that the transformative change continues to benefit medical education across the world.

## Take Home Messages

The COVID-19 pandemic has been a major disruptive change to medical education across the world and the use of technology has been rapidly and innovatively used in an attempt to maintain teaching and learning. When the pandemic resolves, transformative change is likely to occur in the way that technology will be used in medical education, especially with the integration of emergent technology. There are significant benefits to this transformative change but there are important challenges that need to be addressed if the future and continuing use of technology in medical education is to be effective and have a positive impact on both educators and students across the world.

## Notes On Contributors

Poh Sun Goh, MBBS, FRCR, FAMS, MHPE, FAMEE, is an Associate Professor and Senior Consultant Radiologist at the Yong Loo Lin School of Medicine, National University of Singapore, and National University Hospital, Singapore. He is a graduate of the Maastricht MHPE program, a member of the AMEE TEL committee, and a Fellow of AMEE. ORCiD: https://orcid.org/0000-0002-1531-2053

John Sandars MB ChB (Hons), MSc, MD, MRCP, MRCGP, FAcadMEd, CertEd, FHEA is Professor of Medical Education at Edge Hill University Medical School, Ormskirk, UK, and is Co-Chair of the AMEE Technology Enhanced Learning Committee. ORCiD: https://orcid.org/0000-0003-3930-387X

## Declarations

The author has declared that there are no conflicts of interest.

## Ethics Statement

This is a Personal Opinion piece and does not require Ethics Approval.

## External Funding

This article has not had any External Funding
