# Supplementary material for: A vision of the use of technology in medical education after the COVID-19 pandemic
Source: MedEdPublish (2016). 2020 Mar 26;9:49. [Version 1] doi: 10.15694/mep.2020.000049.1 (PMC10697445; doi:10.15694/mep.2020.000049.1)
Supplement: Peer review history

# Reviewer report: Reviewer response for version 1

**DOI:** 10.21956/mep.20027.r30587

**Published:** 2020-04-29

**Authors:** Samar Ahmed

**Affiliations:** 1 Ain Shams University Faculty of Medicine

This review has been migrated. The reviewer awarded 4 stars out of 5 The article is a combination of a status analysis post COVID pandemic and a projection into the future of what is to come after the situation is over. I likes a lit the framework and think it is useful in documenting the situation but i believe more insight into the future sustainable gains if this chaos is needed

Reviewer Expertise:

NA

No decision status is available

# Reviewer report: Reviewer response for version 1

**DOI:** 10.21956/mep.20027.r30595

**Published:** 2020-04-17

**Authors:** Judy McKimm

**Affiliations:** 1 Swansea University

This review has been migrated. The reviewer awarded 5 stars out of 5 I read this article by two experts in TEL with interest and found the framework very helpful indeed. It veered around a bit from being an opinion piece to a position paper with recommendations, and I think defining the focus and structure a bit more clearly would help.However, this is just my view and I thought it was excellent with some good signposting to helpful resources as well. There’s lots more literature on change management such as transition curves, etc and the leadership literature on collaboration etc that might be considered too.I also agree with other reviewers and the authors about Impact of the length of the lockdowns on the normalisation. At the moment we’re still a bit in the excited crisis management stage and we need to shift along the curve so all this learning and potential innovation and transformation isn’t lost A great paper, thank you

Reviewer Expertise:

NA

No decision status is available

# Reviewer report: Reviewer response for version 1

**DOI:** 10.21956/mep.20027.r30594

**Published:** 2020-04-07

**Authors:** P Ravi Shankar

**Affiliations:** 1 American International Medical University

This review has been migrated. The reviewer awarded 4 stars out of 5 This is an interesting article on the use of technology in medical education after the COVID-19 pandemic. As mentioned by the authors the duration and extent of the disruption can influence the possible scenario of medical education. Emergent technology is becoming cheaper and more widely available and today most can be accessed using a smartphone or a tablet. One of the important issues will be the cost of the created content and what may be considered as a fair price for the developers. If it become too high then many educators in developing nations may not be able to access the same. This is another in a series of manuscripts in MedEdPublish on the COVID-19 pandemic and its effects on different aspects of medical education.

Reviewer Expertise:

NA

No decision status is available

# Reviewer report: Reviewer response for version 1

**DOI:** 10.21956/mep.20027.r30591

**Published:** 2020-04-05

**Authors:** Shaoting Feng

**Affiliations:** 1 The First Affiliated Hospital of Sun Yat-sen University

This review has been migrated. The reviewer awarded 5 stars out of 5 Interesting opinion on the use of technology in medical education after the COVID-19 pandemic with rich examples and data! It enlightens us to think deeply about the impact of the COVID-19 pandemic on medical education.

Reviewer Expertise:

NA

No decision status is available

# Reviewer report: Reviewer response for version 1

**DOI:** 10.21956/mep.20027.r30593

**Published:** 2020-04-02

**Authors:** Deb Halder

This review has been migrated. The reviewer awarded 5 stars out of 5 Note on Abstract and IntroductionThe abstract stated the thesis statement of the initiated work except explaining the design of the paper though the very last clause of the abstract “The benefits and challenges of the use of technology in medical education are discussed with the intention of informing all providers on how the changes after the pandemic can have a positive impact on both educators and students across the world” provided the trajectory of constructions of the paper.Introduction should initiate a thesis statement along with the explanation of the scope of ‘technology use’, ‘medical education’, ‘covid-19’ and ‘the vision’ on all these progress and trajectory.The Body of DiscussionThe opinion writing is argumentative and is constructed with examples and researched data. The authors have been tailored to make this conception convincing across the continuum of medical education by providing recent references.Note on Benefits and Challenges of changes:The paper has been neutrally justified with the address of benefits and challenges of the envisaged trajectory of distribution of medical education. But the later portions of both the paragraphs have introduced recommendation; for example, the following statement “Technology that is appropriate to the local contexts, with lower bandwidth cellular and online networks, will need to be considered and international collaboration between medical schools will need to be developed” should have been superimposed at the place of recommendation. Note on Take Home messages and ConclusionThe paper has not directly introduced any discussion on conclusion where the thesis statement of the paper becomes iterative and approved. This paper supposes to fail in this case; Take home messages has been concluding remarks of the paper and that’s why; the recommendation is underestimated. But irrespective of these constructional pitfalls, the introduced idea is constructive, adoptable and it claims novelty. The theme is clear and convincing.

Reviewer Expertise:

NA

No decision status is available

# Reviewer report: Reviewer response for version 1

**DOI:** 10.21956/mep.20027.r30590

**Published:** 2020-03-27

**Authors:** Kieran Walsh

**Affiliations:** 1 BMJ

This review has been migrated. The reviewer awarded 5 stars out of 5 This is an excellent article outlining trends in technology in medical education and how the covid 19 pandemic will affect these trends. The authors are very likely to be right that things will not go back to the way they were before. But how things will change is hard to predict. Technology enhanced learning has been becoming normalised for some time now – it is just another way for students and doctors to learn. Students and doctors often say they use online resources to stay updated. But the pace of change of the knowledge base around covid 19 has been unprecedented. Learners cannot read all of it or stay across all of it. Point of care learning will be needed as a result. This will be especially important for those final year students who are now rapidly preparing for actual practice. It would be interesting to hear the views of the authors and others about technology enhanced learning and a rapidly changing knowledge base.

Reviewer Expertise:

NA

No decision status is available

# Reviewer report: Reviewer response for version 1

**DOI:** 10.21956/mep.20027.r30588

**Published:** 2020-03-26

**Authors:** Manish Khazane

**Affiliations:** 1 SRM UNIVERSITY,RAMAPURAM,CHENNAI.

This review has been migrated. The reviewer awarded 4 stars out of 5 The authors have aptly putforth their views in the present article about the aid by technology in medical education. In the coming months, with a great hope of positivity, we can believe that medical education and the whole aim of equipping medical personels for the future can be achieved more prominently.The spread of the COVID-19 has brought a stark reality to the human kind that change is constant. And this should spring us back in action with better ways of teaching, learning and treating our patients. The ubiquitous availability of video and audio accessories in all our techologies including laptops, mobile phones has eased this difficult phase.Indeed, it is essential to assimilate and plan a new way of reaching out to all the concerned individuals.

Reviewer Expertise:

NA

No decision status is available

# Reviewer report: Reviewer response for version 1

**DOI:** 10.21956/mep.20027.r30589

**Published:** 2020-03-26

**Authors:** Trevor Gibbs

**Affiliations:** 1 AMEE

This review has been migrated. The reviewer awarded 5 stars out of 5 If anything good can come out of the Covid-19 pandemic it is the change that will occur in health professions education ( and education in general) and the emergence of technology -enhanced learning, in all its forms. As someone whose children call a digital dinosaur when it comes to IT, I found this paper very easy to read and an excellent review of both the present and potential future situations re TEL.I strongly recommend it to all those involved in curriculum planning.

Reviewer Expertise:

NA

No decision status is available

# Reviewer report: Reviewer response for version 1

**DOI:** 10.21956/mep.20027.r30592

**Published:** 2020-03-26

**Authors:** Trudie Roberts

**Affiliations:** 1 Leeds Institute of Medical Education

This review has been migrated. The reviewer awarded 4 stars out of 5 An interesting opinion piece from two authors with a major interest and understanding of the area of technology supporting learning. It would be useful to situate this paper with others where the current COVID situation has meant that educators have used technology in practice to over come difficulties for example the recent piece by Judy McKimm and colleagues: Manuscript ID: 2936Article title: Health Professions' Educators' Adaptation to Rapidly Changing Circumstances: The Ottawa 2020 Conference Experience

Reviewer Expertise:

NA

No decision status is available
